# Gut dysbiosis and brain cancer: exploring the emerging role of sweeteners and food additives

**DOI:** 10.3389/frmbi.2026.1960004

**Published:** 2026-10-27

**Authors:** Maryam Sohail, Aiza Tanveer Hassan, Moomena Gulzar, Noor Numan, Alice Njolke Mafe, Dietrich Büsselberg

**Affiliations:** 1 Weill Cornell Medicine-Qatar, Education City, Qatar Foundation, Doha, Qatar; 2 Department of Biological Sciences, Faculty of Sciences, Taraba State University, Jalingo, Taraba State, Nigeria

**Keywords:** artificial sweeteners, dysbiosis, food additives, glioblastoma, gut microbiota, gut–brain axis, short-chain fatty acids, tumor microenvironment

## Abstract

Glioblastoma (GBM) remains the most aggressive primary brain malignancy, with a median survival of approximately 15 months, and its rising incidence has increased interest in modifiable dietary risk factors. Despite growing evidence that food additives disrupt gut microbial ecology and that gut dysbiosis influences neuro-oncological pathways, no previous review has systematically integrated these findings through the gut–brain axis. This narrative review synthesizes evidence from studies on food additives, gut microbiome, and glioblastoma to identify shared mechanistic pathways. Across five additive categories (artificial sweeteners, food colorants, preservatives, nanoparticles, and emulsifiers), a common dysbiotic signature emerged, characterized by depletion of short-chain fatty acid-producing bacteria, increased lipopolysaccharide (LPS) production, disruption of tryptophan metabolism, and impairment of intestinal and blood–brain barrier integrity. These changes converged on neuroinflammatory, immunosuppressive, and oncogenic pathways, including NF-κB, PI3K/AKT, and m^6^A-mediated epigenetic reprogramming. Among all additives reviewed, aspartame provided the most complete hypothesized mechanistic sequence linking gut dysbiosis, epithelial barrier dysfunction, and m^6^A-mediated upregulation of glioblastoma-associated genes, highlighting a plausible microbiome–epigenetic axis that warrants further investigation. These findings position food additives as biologically active modulators of the gut–brain axis with potential relevance to glioblastoma pathogenesis and provide a mechanistic framework to guide future microbiome-informed prevention, biomarker discovery, and therapeutic research.

## Graphical Abstract

**Figure:**
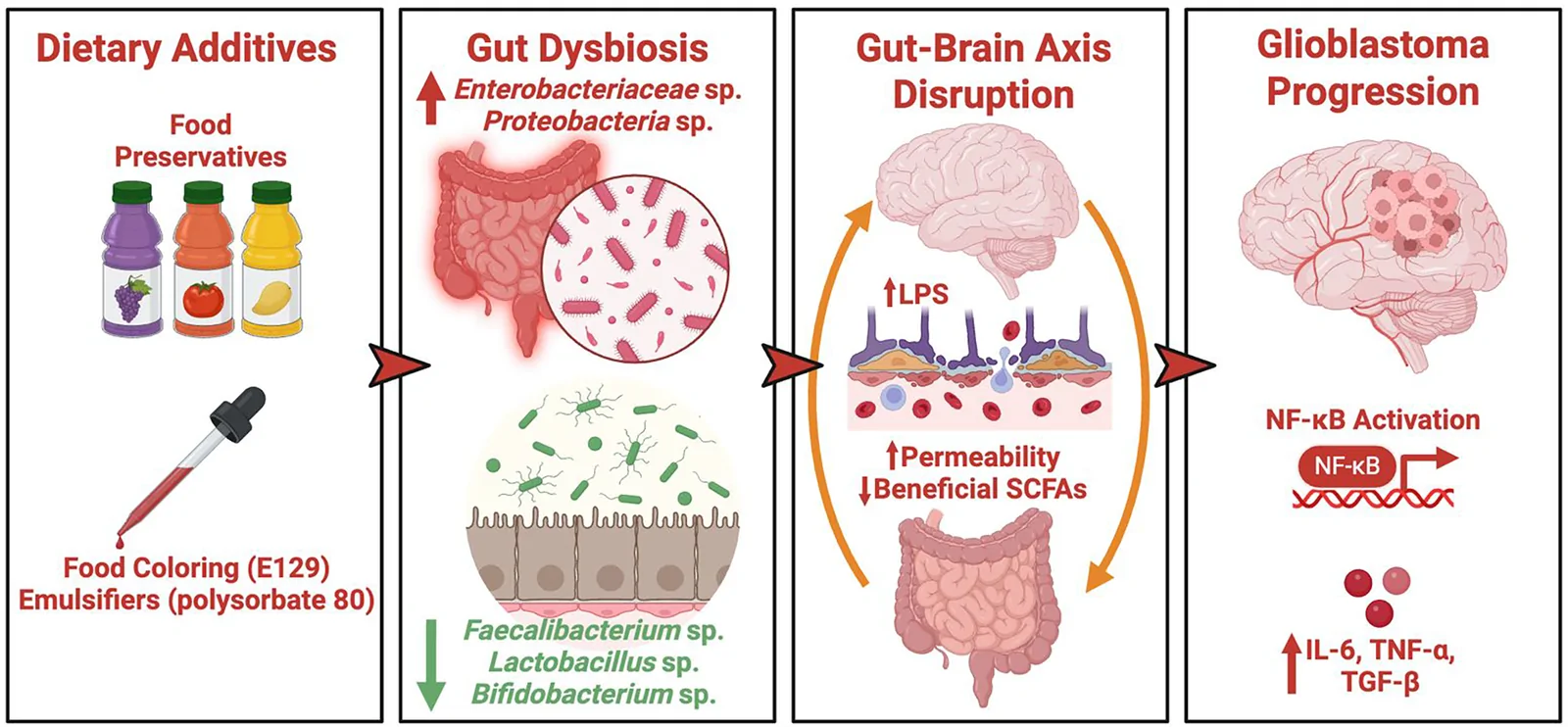
Created in BioRender. Busselberg, D. (2026). https://BioRender.com/tvw4j5m.

## 1 Introduction

Glioblastoma (GBM) is the most common and aggressive primary malignant brain tumor, accounting for 14.5% of all central nervous system tumors and 48.6% of malignant CNS tumors (Grochans et al., 2022). Despite multimodal treatment involving maximal safe surgical resection, concurrent radiotherapy, and temozolomide chemotherapy, the median overall survival remains approximately 15 months, with a 5-year survival rate of only 5% (Tamimi et al., 2017). Moreover, recurrence is near-universal, driven by the tumor’s extensive genetic heterogeneity, invasive growth patterns, and resistance to both conventional and emerging immunotherapies (Królikowska et al., 2025). Epidemiological data from multiple global regions indicate a consistent rise in GBM incidence rates over recent decades (Grech et al., 2020), a trend that cannot be fully accounted for by improvements in diagnostic technology alone and that has prompted increasing interest in modifiable environmental and dietary contributors to tumor development.

In parallel with rising GBM incidence, global dietary patterns have undergone profound transformation. The widespread adoption of processed and ultra-processed foods, which now constitute over 60% of daily caloric intake in several high-income populations, has dramatically increased human exposure to synthetic food additives (Singh et al., 2024). The scale of this exposure is further complicated by substantial gaps in premarket safety oversight. The Food and Drug Administration (FDA) allows the food industry to self-determine whether a new ingredient is Generally Recognized as Safe (GRAS) using safety data that the agency itself never reviews, and currently has no formal process for reassessing food additives or GRAS substances already present in the food supply (Pomeranz et al., 2024). This self-affirmation pathway is not a rarely used provision. Of 451 GRAS safety notifications voluntarily submitted to the FDA between 1997 and 2012, 22.4% were determined safe by an employee of the manufacturer itself, and 13.3% by an employee of a consulting firm the manufacturer selected; none were reviewed by an independent third party (Conflicts of Interest in Approvals of Additives to Food Determined to be Generally Recognized as Safe: Out of Balance | Ethics | JAMA Internal Medicine | JAMA Network). A subsequent study of GRAS notices filed between 2015 and 2020 found a similar problem persisting, in which seven individuals alone occupied nearly half of all available GRAS review-panel positions, and no panel was found to comply with the FDA’s own conflict-of-interest guidance (Out of Balance: Conflicts of Interest Persist in Food Chemicals Determined to be Generally Recognized as Safe | Environmental Health | Springer Nature Link). Consequently, while the additive categories reviewed here carry regulatory approval for general consumption, none has been evaluated for its capacity to disturb the gut microbiome as a potential upstream driver of CNS pathology, and none has been assessed specifically in relation to glioblastoma risk.

Over the past decade, the gut–brain axis has emerged as a critical bidirectional communication network linking gastrointestinal function to central nervous system physiology. Signaling between the gut microbiota and the brain occurs through three principal routes: neural transmission via the vagus nerve and enteric nervous system, immune-mediated signaling through cytokines and pattern recognition receptors, and metabolic communication through microbial metabolites including short-chain fatty acids (SCFAs), tryptophan derivatives, lipopolysaccharides (LPS), and secondary bile acids (Loh et al., 2024; You et al., 2024; Ganesh et al., 2025). Disruption of this axis through gut dysbiosis has been implicated in the pathogenesis of multiple neurological disorders, and accumulating evidence now suggests that these same mechanisms may extend to neuro-oncological disease (He et al., 2024). Specifically, dysbiosis-driven alterations in microbial metabolite production—particularly SCFA depletion, LPS elevation, and tryptophan–kynurenine pathway disruption—have been shown to compromise blood–brain barrier integrity, promote neuroinflammation, and modulate immune checkpoint signaling within the tumor microenvironment (Mafe and Büsselberg, 2025).

Prior reviews have largely studied these two bodies of evidence independently. Reviews of food additives and gut health are typically bounded within microbial composition and local gastrointestinal outcomes, without extending to distal organ systems (Singh et al., 2024). Conversely, reviews of the gut–brain axis in neuro-oncology have concentrated on characterizing microbiome differences in patients already diagnosed with brain tumors, or on general neuroinflammatory mechanisms, without addressing which dietary exposures might drive the initiating dysbiosis (He et al., 2024; Loh et al., 2024; You et al., 2024; Mafe and Büsselberg, 2025). This fragmentation makes it difficult to assess whether food additive-induced dysbiosis is a plausible, testable contributor to GBM risk, or to highlight which mechanistic links most urgently need prospective human investigation.

This review fills these gaps by bringing together evidence from studies on food additives and the gut microbiome, the gut–brain axis, and GBM into a single mechanistic framework. Unlike previous reviews that discuss food additives, gut dysbiosis, or GBM as separate topics, this review connects these processes step by step: from dietary additive exposure to changes in the gut microbiome, altered microbial metabolite production, gut–brain axis signaling, blood–brain barrier dysfunction, and ultimately the activation of pro-tumor and immunosuppressive pathways within the GBM tumor microenvironment.

## 2 Methodology

This narrative review synthesizes current evidence on the relationship between food additives, gut microbiome dysbiosis, and GBM through the gut–brain axis. Scientific articles were retrieved from PubMed, Scopus, ScienceDirect, and Google Scholar. The search was conducted in two stages: the first identified studies on food additives and gut dysbiosis using combinations of keywords including food additives, artificial sweeteners, sucralose, aspartame, saccharin, xylitol, preservatives, emulsifiers, food colorants, nanoparticles, gut microbiota, gut microbiome, intestinal microbiota, dysbiosis, and fecal microbiota; the second identified studies on the gut microbiome and GBM using combinations of *gut microbiota*, *gut microbiome*, *dysbiosis*, *fecal microbiota transplantation*, *glioblastoma*, *glioma*, *brain tumor*, *brain cancer*, and *brain neoplasms*. Studies published in English between 2015 and 2026 were eligible if they were peer-reviewed original research articles, including clinical, preclinical, Mendelian randomization, and computational studies. Review articles, editorials, non-English publications, and studies lacking methodological transparency were excluded from the primary evidence synthesis, although reviews were consulted for contextual background where primary evidence was limited. Duplicates were removed using Zotero reference management software, and titles, abstracts, and full texts were screened for relevance. This is a narrative review employing a structured but non-systematic literature search; no formal PRISMA protocol was followed, and the synthesis is qualitative rather than quantitative. Throughout this review, “glioblastoma” (GBM) refers specifically to WHO grade IV glioma, the most aggressive subtype, while “glioma” is used as a broader term encompassing all glial-origin brain tumors, including lower-grade variants. Where individual studies investigated glioma models or populations without grade-specific stratification, the term “glioma” is retained to accurately reflect the original evidence. For each primary study included in the synthesis, we recorded the experimental model, the exposure conditions (substance, dose, route, and duration), and the methods used to measure the microbiota and its metabolites. These are set out in the **Supplementary Table**. The distinction matters because 16S rRNA gene sequencing, shotgun metagenomics, targeted qPCR, and culture-based assays produce different kinds of data, and several studies have predicted metabolic pathways computationally rather than directly measuring metabolites. Since the included studies differ in model, exposure, and analytical platform, each finding is interpreted considering the design that produced it, and quantitative comparisons between studies are avoided. Null and conflicting results are also recorded in the **Supplementary Table** alongside positive ones.

## 3 Thematic analysis

The results of this narrative review are organized into two linked sections. Section 3.1 presents the evidence for food additive-induced gut dysbiosis, demonstrating how five major categories of dietary additives converge on a shared pattern of microbial disruption. Section 3.2 follows the metabolite classes through which additive-induced dysbiosis communicates with the central nervous system via the gut–brain axis. Rather than considering each additive independently, this review emphasizes the convergence of apparently unrelated chemical classes on common microbial, metabolic, and barrier-associated pathways. This systems-level synthesis provides the conceptual basis for the downstream gut–brain axis mechanisms discussed later.

### 3.1 Food additives and gut microbial disruption

A structured examination of the sweeteners and additives in **Table 1** identified 23 studies, of which 22 span five major categories of dietary additives: artificial sweeteners, food colorants, preservatives, nanoparticles, and emulsifiers or mixed-additive formulations. Each independently disrupted gut microbial composition and metabolic function. Despite this diversity, the underlying evidence converges on a consistent downstream signature, detailed in Section 3.1.6, alongside one additional study on the mechanisms involved in dietary sugar. The **Supplementary Table** gives the model, exposure conditions, and analytical method for every study cited in this section and in Sections 3.2 to 3.4, together with any null or conflicting results. These studies range from pure bacterial culture and closed fermentation to avian embryo, rodent, and porcine models, and to human trials, with exposure durations varying widely. The convergence described below is therefore in the direction of the effect rather than in its magnitude.

**Table 1:** Summary of food additives included in the review.

Additive category	Representative compounds	Number of studies	Main microbial alterations	Main metabolites altered	Principal biological consequence	Reference
Artificial sweeteners	Sucralose, saccharin, aspartame, xylitol, acesulfame-K, stevia, NHDC, cyclamate, neotame	13	*↓ Roseburia*, Lachnospiraceae, *Bifidobacterium* sp., *Lactobacillus* sp., *Bacteroides*; *↑ Turicibacter* sp., *Ruminococcus* sp., *Akkermansia* sp., Enterobacteriaceae (xylitol reverses direction)	↑ Quinolinic acid, ROS, endotoxin; ↓ kynurenic acid, L-tryptophan; ↑ SCFAs (xylitol only)	Tight-junction loss, colitis susceptibility, colitis-associated CRC risk; one non-microbiome route via direct protein binding	(Bian et al., 2017a; 2017b; Uebanso et al., 2017; Ahmad et al., 2020; Li et al., 2020; Nettleton et al., 2020; Shil et al., 2020; Guo et al., 2021; Xiang et al., 2021; Mendoza-Martínez et al., 2022; Zani et al., 2023; Bellanco et al., 2024; Xie et al., 2025)
Food colorants	Allura Red AC (Red 40), Sunset Yellow (Yellow 6)	2	Microbiota-dependent ↑ serotonin production; ↑ dye-metabolizing commensals (*Bacteroides ovatus*, *Enterococcus faecalis*)	↑ Serotonin (5-HT); ANSA-Na (genotoxic azo-reduction intermediate)	↓ ZO-1, ↑ colitis susceptibility, IL-23-dependent CD4^+^ T-cell activation	(He et al., 2021; Kwon et al., 2022)
Preservatives	Sodium benzoate, potassium sorbate, sodium nitrite, sulfites, nisin (E234), BHA, EDTA	4	↓ *Clostridium tyrobutyricum*, *Lactobacillus* sp., *Bifidobacterium* sp.; *↑ Enterococcus faecalis*, brief Gram-negative expansion	↓ Acetate, butyrate, branched-chain fatty acids (BCFA), cecal acetate (EDTA)	Dysbiosis skewed toward pro-inflammatory/colitogenic taxa; sex-specific host metabolic effects with EDTA	(Irwin et al., 2017; Hrncirova et al., 2019; O’Reilly et al., 2023; Schell et al., 2025)
Nanoparticles	TiO_2_ (E171), SiO_2_, ZnO, Fe_2_O_3_, silver nanoparticles/nanowires	2	↓ *Clostridium* sp., *E. coli* (TiO_2_ vs. ZnO/Fe_2_O_3_); ↓ Gram-negative bacteria, overall diversity (silver, short-term only)	↑ 1H-indole-3-carboxylic acid, serotonin (silver, long-term, post-recovery)	Impaired brush-border membrane functionality; persistent metabolic reprogramming despite apparent microbiota recovery by day 28	(Cheng et al., 2023; Wang et al., 2023)
Emulsifiers/mixed additives	Maltodextrin, CMC, polysorbate-80, carrageenan-κ, cinnamaldehyde, sodium benzoate, sodium sulfite, TiO_2_, aspartame, sucralose, stevia	1	↑ *B. coccoides* group, *Bifidobacterium* sp. (maltodextrin + aspartame); ↑ Shannon α-diversity (stevia); ↓ *Faecalibacterium* sp., *Firmicutes* (cinnamaldehyde)	↑ Acetic acid, propionic acid, total SCFAs (maltodextrin/aspartame); ↓ butyrate, BCFAs (cinnamaldehyde, sodium sulfite)	Additive-specific, non-uniform microbiota shifts; cinnamaldehyde most disruptive, only stevia raising diversity	(Gerasimidis et al., 2020)
Dietary sugar (outside formal categories)	High-fructose diet	1	↑ Proteobacteria, Deferribacteraceae, Helicobacteraceae; *↓* Bacteroidetes	↓ Acetate, propionate, butyrate, total SCFAs	Colonic NLRP6 inflammasome dysfunction, hippocampal neuroinflammation and neuronal loss; reversed by SCFA/pioglitazone supplementation	(Li et al., 2019)

↑, increased abundance or level; ↓, decreased abundance or level, relative to control or unexposed conditions in the cited studies.

Although the additives reviewed differ substantially in chemical structure and intended technological function, their biological effects display remarkable convergence. Across multiple experimental models, recurrent alterations were observed in microbial composition, metabolite production, epithelial barrier integrity, and inflammatory signaling, suggesting that gut dysbiosis represents a common biological response rather than an additive-specific phenomenon.

#### 3.1.1 Artificial sweeteners

Artificial sweeteners constituted the most extensively investigated class of additives in the available literature (13 of 23 studies) and therefore provide the strongest evidence linking dietary additives to microbiome remodeling, with sucralose in particular emerging as the most extensively documented additive implicated in gut barrier-associated immune disruption. Chronic exposure to sucralose disrupted the tryptophan–kynurenine pathway, elevating quinolinic acid while depleting kynurenic acid and L-tryptophan. Furthermore, expansion of bacteria such as *Turicibacter* sp., *Ruminococcus* sp., and *Akkermansia* sp. occurred alongside the depletion of *Roseburia* sp. and other Lachnospiraceae members. These dysbiotic changes co-occurred with an increase in hepatic iNOS and MMP-2 expression, consistent with a pro-inflammatory liver phenotype (Bian et al., 2017a). In parallel, sucralose was found to activate T1R3 sweet receptors, reducing claudin-3 expression and disrupting tight-junction organization, thereby increasing epithelial permeability. The presence of aspartame induced reactive oxygen species (ROS) and reduced epithelial cell viability (Shil et al., 2020). *In vivo*, sucralose exposure increased susceptibility to dextran-sulfate-sodium (DSS)-induced colitis, elevating IL-1β, IL-10, TRAF6, TNF-α, and TLR4 while downregulating mucosal occludin, claudin-1, and claudin-4, alongside expansion of *Firmicutes*-associated taxa (e.g., *Peptostreptococcus stomatis*, *Clostridium symbiosum*) and depletion of Proteobacteria and *Bifidobacteria* (Guo et al., 2021). A companion study, using the same exposure framework, linked this inflammatory signature and barrier disruption to increased colitis-associated colorectal cancer risk in a murine model (Li et al., 2020). These studies indicate that sucralose influences multiple biological layers simultaneously, including microbial ecology, epithelial integrity, immune activation, and microbial metabolism, making it the most comprehensively characterized additive within the current evidence base.

Saccharin exposure produced a distinct but convergent outcome, altering gut microbiota composition and bacterial functional gene content toward pro-inflammatory, increased lipopolysaccharide (LPS) biosynthesis and bacterial toxins, with expansion of *Sporosarcina* sp., *Oscillospira* sp., and *Akkermansia* sp. and depletion of *Ruminococcus* sp., *Anaerostipes* sp., and *Dorea* sp.; this shift was accompanied by increased hepatic TNF-α and iNOS and depletion of the anti-inflammatory metabolites equol, palmitoylethanolamide, and linoleoylethanolamide (Bian et al., 2017b). Non-caloric sweetener (NCS) mixtures, composed predominantly of sucralose with aspartame, acesulfame-K, and saccharin, produced shifts toward Enterobacteriaceae, *Clostridium leptum*, and Deltaproteobacteria, with simultaneous depletion of *Bifidobacterium* sp., *Lactobacillus* sp., and *Bacteroides* sp (Bian et al., 2017b). An NCS-free diet was found to alleviate abdominal pain, post-prandial discomfort, early satiety, and epigastric pain, while the NCS diet increased diarrhea, constipation, and postprandial discomfort (Mendoza-Martínez et al., 2022).

Importantly, not all sweeteners produced uniformly detrimental outcomes, highlighting the complexity of additive–microbiome interactions. Xylitol presented a protective profile across three studies. Unlike other sweeteners, xylitol increased colonic SCFA production, including acetate, propionate, and butyrate via cross-feeding interactions, and expanded *Bifidobacterium* sp., *Lactobacillus* sp., and *Erysipelotrichaceae* while shifting the Firmicutes:Bacteroidetes ratio upward (Uebanso et al., 2017; Xiang et al., 2021). One study found xylitol’s butyrogenic impact to be accompanied by elevated ammonium, a compound with recognized neurotoxic potential; the authors characterized the net neuro-oncological impact of this as unresolved (Bellanco et al., 2024). These contrasting findings indicate that microbial responses are additive-specific and highly context-dependent, emphasizing that biological effects cannot simply be generalized across all non-nutritive sweeteners.

Not all evidence points toward sweetener-induced dysbiosis, and a route to glioma risk was found that bypasses the microbiome altogether. In healthy human adults, a 14-day randomized double-blind crossover trial found that aspartame and sucralose produced no measurable change in gut microbiota composition, diversity, community structure, or SCFA output (Ahmad et al., 2020). Separately, an *in silico* network pharmacology analysis predicted that aspartame, acesulfame, sucralose, neohesperidin dihydrochalcone, cyclamate, neotame, and saccharin bind core cancer-associated protein targets, including components of the *EGFR* tyrosine-kinase-inhibitor-resistance and PI3K-Akt signaling pathways. Targets were identified from prediction databases (CTD, STITCH, Super-PRED, Swiss Target Prediction, SEA, PharmMapper), and binding was assessed computationally by molecular docking in AutoDock Vina, with interactions defined as strong at affinity scores ≤ −5.0 kcal/mol; associations with low-grade glioma derive from univariate Cox regression on target-gene expression in TCGA, rather than from measured sweetener exposure. No gut microbiota were characterized, and no experimental validation was performed (Xie et al., 2025). Finally, a maternal-exposure study found that low-dose aspartame or stevia consumption during an obesogenic diet altered gut microbiota composition and SCFA output in rat dams, elevating cecal propionate, butyrate, and isobutyrate, while increasing *Porphyromonadaceae* and *Clostridium leptum* and decreasing *Enterococcaceae*, *Enterococcus* sp., and *Parasutterella*; these maternal shifts were accompanied by altered offspring gut microbiota, increased offspring adiposity, impaired glucose tolerance, and lasting changes in offspring mesolimbic dopamine reward-pathway gene expression, although the outcomes measured were metabolic and behavioral rather than oncologic (Nettleton et al., 2020). Two further results qualify this pattern. In the Allura Red AC study (Kwon et al., 2022), intermittent rather than daily exposure over 12 weeks left colitis susceptibility unchanged, and the dye raised colonic serotonin in germ-free mice as well as colonized ones, so part of the effect does not depend on the microbiota. Zani et al. (2023) gave sucralose at doses equivalent to the acceptable daily intake and found no consistent change in bacterial species at either 2 or 12 weeks. In that study, sucralose reduced colitis severity and hyperglycemia while increasing tumor growth, acting on T cells rather than through the microbiome. This runs counter to the mechanism proposed elsewhere in this review and suggests that sucralose may affect tumor immunity via a route that does not involve the gut microbiota (see **Supplementary Table**).

#### 3.1.2 Food colorants

Allura Red AC (FD&C Red 40) increased colonic serotonin (5-HT) production through a microbiota-dependent and microbiota-independent mechanism, which in turn elevated pro-inflammatory cytokines (IL-1β, IL-6, TNF-α), downregulated the tight-junction gene ZO-1 (*Tjp1*), and increased susceptibility to experimental colitis; cecal microbiota transfer from Red 40-exposed mice was sufficient to worsen experimental colitis in germ-free recipients, demonstrating a causal dysbiotic phenotype (Kwon et al., 2022). In a combined exposure study in mice with dysregulated IL-23 expression, ANSA-Na triggered IL-23-dependent CD4^+^ T-cell activation and IBD-like colitis, an effect requiring the presence of dye-metabolizing commensals (He et al., 2021). Together, these studies demonstrate that food colorants exert biological effects that extend beyond direct host toxicity and require active participation of the gut microbiota to generate inflammatory and potentially genotoxic metabolites.

#### 3.1.3 Preservatives

Sodium benzoate, potassium sorbate, and sodium nitrite suppressed SCFA-producing and anti-inflammatory taxa, including *Clostridium tyrobutyricum*, *Lactobacillus paracasei*, *Bacteroidales*, and *Bifidobacterium*, while pro-inflammatory or colitogenic strains such as *Enterococcus faecalis* and *Bacteroides thetaiotaomicron* showed greater resistance, producing a dysbiosis skewed toward pro-inflammatory taxa (Hrncirova et al., 2019). Sulfites (sodium bisulfite, sodium sulfite) inhibited the growth of beneficial lactic acid bacteria *Lactobacillus casei*, *Lactobacillus plantarum*, *Lactobacillus rhamnosus*, and *Streptococcus thermophilus* within hours of exposure, at concentrations regarded as safe under current food-additive regulations (Irwin et al., 2017). Nisin (E234), a bacteriocin preservative, decreased acetate, butyrate, and BCFAs while promoting transient expansion of Gram-negative taxa such as *Escherichia coli* during treatment, an effect that was largely reversible after cessation (O’Reilly et al., 2023). A mixed-preservative study of acetic acid, butylated hydroxyanisole (BHA), EDTA, and sodium sulfite revealed antimicrobial reorganization of the gut microbiota with sex-specific host metabolic consequences, including reduced cecal acetate and a 32% reduction in fat gain in female mice following early-life EDTA exposure, with no comparable effect in males (Schell et al., 2025). Unlike sweeteners that primarily altered microbial metabolism, preservatives preferentially reshaped microbial ecology through selective antimicrobial activity, thereby favoring inflammation-associated taxa. The **Supplementary Table** shows how narrow the evidence is in this category. The benzoate, sorbate, nitrite, and sulfite results all come from pure bacterial culture over a few hours, with no host and no microbial community. The nisin study was conducted in post-weaning piglets over 4 days, and its effects were reversed once dosing stopped. None of these studies measured a host inflammatory or barrier endpoint, and none can be set directly against the multimonth rodent sweetener studies described above.

#### 3.1.4 Nanoparticles

Food-grade metal oxide nanoparticles (TiO_2_/E171, SiO_2_, ZnO, Fe_2_O_3_) produced species- and particle-specific inhibition patterns and impaired intestinal brush-border membrane functionality, with TiO_2_ and SiO_2_ being associated with the greatest reductions in *Clostridium* sp. and *E. coli* relative to ZnO and Fe_2_O_3_ exposure (Cheng et al., 2023). Silver nanomaterials (nanoparticles and nanowires) produced short-term inhibition of Gram-negative bacteria and reduced microbial diversity, which recovered by 28 days; however, this apparent structural recovery masked persistent metabolic reprogramming, with long-term increases in 1H-indole-3-carboxylic acid and serotonin in both gut and blood, indicating durable disruption of tryptophan metabolism despite recovery of composition (Wang et al., 2023). These findings suggest that nanoparticle-associated dysbiosis may persist functionally even after apparent restoration of microbial composition, emphasizing the importance of metabolomic assessment alongside taxonomic profiling.

#### 3.1.5 Emulsifiers and mixed additives

A study simultaneously testing six additive classes, maltodextrin, carboxymethylcellulose (CMC), polysorbate-80, carrageenan-κ, cinnamaldehyde, sodium benzoate, sodium sulfite, and TiO_2_, alongside the sweeteners aspartame, sucralose, and stevia, found additive-specific, non-uniform shifts in microbiota. Maltodextrin and aspartame co-exposure increased *Bifidobacterium* coccoides and acetic acid production, while sodium sulfite-containing formulations decreased acetate output; cinnamaldehyde produced the most profound overall disruption, reducing Firmicutes sp., *Faecalibacterium* sp., and BCFAs, and stevia was the only additive associated with increased microbial Shannon α-diversity (Gerasimidis et al., 2020). This is the only study in **Table 2** to test additive combinations directly, underscoring how little is known about real-world mixture effects. Given that human dietary exposure almost always involves mixtures rather than isolated additives, this evidence highlights a major knowledge gap that requires future investigation.

**Table 2:** Comparative effects of different food additive classes on gut microbial composition.

Additive	Increased taxa	Decreased taxa	Barrier effect	Inflammatory markers	Overall dysbiotic direction	References
Artificial sweeteners	*Turicibacter* sp., *Ruminococcus* sp., *Akkermansia* sp., *Staphylococcus* sp., *Streptococcus* sp., *Sporosarcina* sp., *Oscillospira* sp., Enterobacteriaceae, *Clostridium leptum*, Deltaproteobacteria, *Bifidobacterium* sp./*Lactobacillus* sp. (xylitol only)	*Roseburia* sp., *Anaerostipes* sp., *Dehalobacterium* sp., *Erysipelotrichaceae*, *Bifidobacterium* sp., *Lactobacillus* sp., *Bacteroides*, *Ruminococcus* sp., *Adlercreutzia* sp., *Dorea* sp. (varies by compound)	↓ Claudin-1/3/4, occludin (T1R3-mediated); ↓ intracellular Ca²^+^ in T cells (immune barrier, not epithelial)	↑ IL-1β, IL-10, TRAF6, TNF-α, TLR4	Predominantly pro-inflammatory; xylitol is the consistent exception (SCFA-raising)	(Li et al., 2019; Shil et al., 2020; Bellanco et al., 2024; Mafe and Büsselberg, 2025; Xie et al., 2025)
Food colorants	*Bacteroides ovatus*, *Enterococcus faecalis* (as dye-metabolizers)	Not individually quantified	↓ ZO-1 (*Tjp1*)	↑ IL-1β, IL-6, TNF-α; IL-23-dependent CD4^+^ activation	Pro-inflammatory/colitogenic	(He et al., 2021; Kwon et al., 2022)
Preservatives	Gram-negative taxa (*E. coli*), transient only (nisin)	*Clostridium tyrobutyricum*, *Lactobacillus* sp., *Bacteroidales*, *Bifidobacterium* sp., *Streptococcus thermophilus*	Not directly assessed	Not directly assessed	Shift favoring pro-inflammatory/colitogenic taxa; largely reversible on cessation (nisin)	(Irwin et al., 2017; Hrncirova et al., 2019; O’Reilly et al., 2023; Schell et al., 2025)
Nanoparticles	Not consistently reported as increased	*Clostridium* sp., *E. coli* (TiO_2_/SiO_2_); Gram-negative bacteria, overall diversity (silver, short-term)	Impaired brush-border membrane function	Not directly assessed	Functional/metabolic disruption persists despite apparent compositional recovery	(Cheng et al., 2023; Wang et al., 2023)
Emulsifiers/mixed additives	*B. coccoides*, *Bifidobacterium* sp., *E. coli*/*Shigella* sp., *Bilophila* (compound-specific)	*Faecalibacterium* sp., Firmicutes, *Bacteroides*/*Prevotella* (compound-specific)	Not directly assessed	Not directly assessed	Additive-specific, non-uniform; most severe with cinnamaldehyde	(Gerasimidis et al., 2020)

↑, increased abundance or level; ↓, decreased abundance or level, relative to control or unexposed conditions in the cited studies.

#### 3.1.6 The recurrent dysbiotic pattern

Cross-referencing the additive studies in this section shows a recurring dysbiotic signature across the four affected categories. At the taxonomic level, this signature is characterized by depletion of SCFA-producing commensals including *Bifidobacterium* (Hrncirova et al., 2019; Mendoza-Martínez et al., 2022), *Lactobacillus* (Irwin et al., 2017; Hrncirova et al., 2019; Mendoza-Martínez et al., 2022), and *Faecalibacterium* sp (Gerasimidis et al., 2020). alongside expansion of Proteobacteria-associated taxa, including Deltaproteobacteria and Enterobacteriaceae (Mendoza-Martínez et al., 2022), and transient Gram-negative expansion (O’Reilly et al., 2023), including Firmicutes-associated shifts in colitis models (Uebanso et al., 2017; Li et al., 2020; Guo et al., 2021).

At the functional level, four metabolite consequences recur across otherwise unrelated additive categories (1): reduced SCFA production, reported with nisin and mixed preservatives (O’Reilly et al., 2023; Schell et al., 2025) (2); elevated LPS or LPS-pathway gene expression, reported with saccharin and with sucralose/aspartame mixtures acting on T1R3 (Bian et al., 2017a; Schell et al., 2025) (3); tryptophan pathway disruption, reported along the kynurenine axis with sucralose and the indole axis with silver nanoparticles (Bian et al., 2017b; Bellanco et al., 2024); and (4) tight-junction loss, reported across sweeteners and colorants alike (Shil et al., 2020; Guo et al., 2021; Kwon et al., 2022). This four-part signature recurs across sweeteners, azo dyes, preservatives, and nanoparticles, additive classes that share no common chemical structure.

This signature is not universal across the studies. A 14-day randomized double-blind crossover trial of aspartame and sucralose in 17 healthy individuals, at 14% and 20% of the acceptable daily intake, respectively, detected no change in microbiota composition or SCFA output (Ahmad et al., 2020), and a separate analysis linked artificial sweeteners to glioma-associated signaling pathways through direct protein binding rather than microbiota disruption (Xie et al., 2025). Both findings are reported here as boundary conditions on the signature described above, not as contradictions of it.

One study outside the formal additive categories reproduced the same sequence in a different dietary exposure. A high-fructose diet reduced fecal acetate, propionate, and butyrate and shifted composition toward Proteobacteria and away from Bacteroidetes in mice; this SCFA depletion was accompanied by colonic NLRP6 inflammasome dysfunction, intestinal barrier impairment, and hippocampal microglial activation and neuronal loss, and each of these outcomes was reversed by SCFA or pioglitazone (PPAR-γ agonist) supplementation, while antibiotic depletion of the microbiota blocked the neuroinflammation but not the neuronal loss (Li et al., 2019). Taken together, the available evidence supports the concept of a recurrent, though non-universal, dysbiotic pattern characterized by depletion of SCFA-producing commensals, disruption of microbial metabolite production, increased epithelial permeability, and activation of inflammatory pathways (summarized in **Figure 1** and **Table 3**). This mechanistic convergence provides the biological foundation for the gut–brain communication pathways explored in the following section.

**Figure 1:**
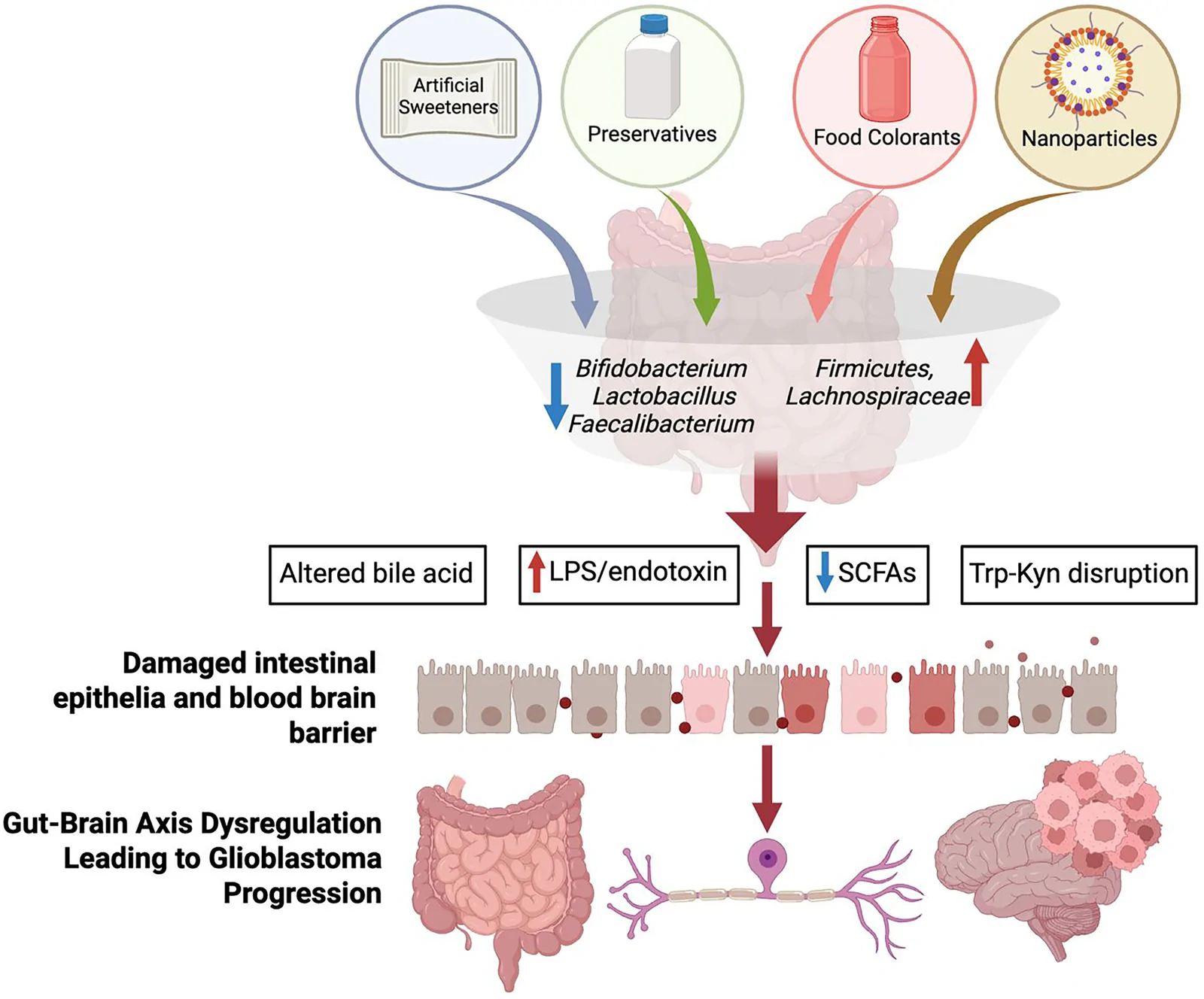
Food additive-induced gut dysbiosis and gut–brain axis disruption: Chronic exposure to dietary additives (artificial sweeteners, preservatives, food colorants, and nanoparticles) drives opposing shifts in gut microbial composition: blue arrows indicate depletion of SCFA-producing taxa (*Bifidobacterium* sp., *Lactobacillus* sp., *Faecalibacterium* sp.), while red arrows indicate expansion of taxa enriched under dysbiotic conditions, including members of Firmicutes and Lachnospiraceae reported in individual studies. These microbial shifts reduce SCFA production, elevate circulating LPS/endotoxin levels, and disrupt bile acid profiles, thereby compromising intestinal epithelial barrier integrity. The resulting increase in gut permeability facilitates translocation of inflammatory mediators into systemic circulation, ultimately dysregulating the gut–brain axis and establishing a neuroinflammatory environment relevant to glioblastoma progression. Created in BioRender. Busselberg, D. (2026). https://BioRender.com/tvw4j5m.

**Table 3:** Recurrent dysbiotic signature across food additives.

Biological feature	Artificial sweeteners	Colorants	Preservatives	Nanoparticles	Mixed additive	Reference
↓ *Bifidobacterium*	✓ (most compounds)	-not assessed	✓	– Not assessed	✗ (↑ instead)	(Hrncirova et al., 2019; Gerasimidis et al., 2020; Mendoza-Martínez et al., 2022; O’Reilly et al., 2023)
↓ *Lactobacillus*	✓	-not assessed	✓	– Not assessed	– Not assessed	(Irwin et al., 2017; Hrncirova et al., 2019; Mendoza-Martínez et al., 2022; O’Reilly et al., 2023)
↓ SCFAs	✗ (xylitol ↑)	-not assessed	✓	– Not assessed	✓ (sulfite/cinnamaldehyde only)	(Gerasimidis et al., 2020; Schell et al., 2025)
↑ LPS	✓ (saccharin, sweetener mix)	-not assessed	– Not assessed	– Not assessed	– Not assessed	(Bian et al., 2017a; Shil et al., 2020)
↑ Cytokines	✓	✓	– Not assessed	– Not assessed	– Not assessed	(Guo et al., 2021; He et al., 2021; Kwon et al., 2022)
Tight junction loss	✓	✓	– Not assessed	✓ (brush-border membrane)	– Not assessed	(Shil et al., 2020; Guo et al., 2021; Kwon et al., 2022; Cheng et al., 2023)

✓, reported in at least one included study for that additive category; ✗, not observed, or opposite direction reported (see parentheses); “not assessed”, not measured in the included studies; ↑, increased; ↓, decreased.

### 3.2 Aspartame case study

Among the additives reviewed in Section 3.1, aspartame is the only compound for which the available evidence, taken together, sketches a hypothesized continuum from dietary exposure through gut dysbiosis, epithelial barrier compromise, epigenetic reprogramming, and glioblastoma-specific molecular consequences. Other additives in this review have evidence specific to either gut dysbiosis alone or downstream gut–brain axis metabolites. This section traces the aspartame-driven continuum and evaluates how far it currently extends.

#### 3.2.1 Evidence for gut microbiome

Aspartame’s microbiome effects have been primarily documented in combination with other sweeteners, rather than in isolation. Non-caloric sweetener mixtures composed predominantly of sucralose with aspartame and acesulfame-K depleted *Bifidobacterium* sp., *Lactobacillus* sp., and *Bacteroides* while expanding Enterobacteriaceae (Mendoza-Martínez et al., 2022). In a six-additive mixture study, aspartame co-exposure with maltodextrin increased *Bifidobacterium*, *B. coccoides*, and acetic acid production, an effect that runs counter to the depletion pattern seen in NCS mixtures (Gerasimidis et al., 2020; Mendoza-Martínez et al., 2022). A maternal-exposure study found that low-dose aspartame consumption during an obesogenic diet altered maternal gut microbiota and cecal SCFA output, with effects transmitted to offspring microbiota and offspring mesolimbic dopamine reward pathway gene expression (Nettleton et al., 2020). This outcome, although not oncogenic, provides evidence for the gut-microbiota-to-brain gene expression link. Surprisingly, a 14-day human trial found that aspartame at regulatory intake levels produced no measurable change in gut microbiota composition, diversity, or SCFA levels (Ahmad et al., 2020). This provides an important condition for aspartame, indicating that microbiome effects, when present, may depend on co-exposure, dose, duration, or host species rather than on a universal, dose-independent property of the compound.

#### 3.2.2 Evidence for barrier disruption

Aspartame co-exposure with sucralose and saccharin induced ROS and reduced intestinal epithelial cell viability in a Caco-2 model (Shil et al., 2020), establishing a plausible route from aspartame exposure to compromised gut barrier integrity, consistent with the tight-junction loss documented across sweeteners generally (Section 3.1.6).

#### 3.2.3 Evidence for glioblastoma

The clearest evidence linking aspartame specifically to glioblastoma comes from a metagenomic and transcriptomic analysis in an intracranial mouse tumor model (Meng et al., 2025). Mice given aspartame in drinking water for 15 days showed a significant decrease in Rikenellaceae abundance relative to controls, alongside a genome-wide shift in m^6^A RNA methylation and mRNA expression in tumor tissue. Three m^6^A-regulated genes (*CDKN1A*, *MYC*, and *TGFB1*) were significantly upregulated in aspartame-exposed tumors, and this pattern was independently validated in human glioblastoma datasets (TCGA and CGGA). Among the genes, *TGFB1* expression was associated with worse survival outcomes according to CGGA, leading the researchers to propose the aspartame–m^6^A–*TGFB1* axis as a candidate mechanism. Notably, the aspartame diet did not significantly affect tumor growth in this model, and the finding is of epigenetic and molecular reprogramming occurring independent of changes in tumor size. These findings identify dietary aspartame as a potential modulator of the glioblastoma epigenome and lay the foundation for future studies to define the molecular mechanisms and causal pathways underlying this relationship.

#### 3.2.4 Evidence from dietary restriction rather than exposure

Independent support for this axis comes from the opposite direction. A 2026 study found that intermittent fasting selectively suppressed *TP53*-subtype glioblastoma in mice through a gut-microbiota-mediated pathway. Fasting was found to reshape the gut microbiome, reduce microbial methionine production, decrease m^6^A methylation, and suppress TGF-β signaling, with this mechanism absent in the *CDKN2A*-subtype model (Lin et al., 2026). The convergence of the two independent studies on the same gut-microbiota–m^6^A–TGF-β signaling axis, with one dietary exposure (aspartame) associated with activation and one dietary restriction (fasting) associated with suppression, is the strongest bidirectional evidence connecting a specific additive to this epigenetic mechanism in glioblastoma. To avoid overstating the similarity, it is necessary to recognize that two studies identify different specific TGF-β family members as their target (*TGFB1* in the aspartame study and *TGFB2* in the fasting study), so rather than being an exact molecular match, the mechanistic overlap is at the pathway level.

#### 3.2.5 Evidence outside glioblastoma

A study in a spontaneous intestinal tumor mouse model found that aspartame exposure at physiologically relevant doses selectively enriched *Akkermansia muciniphila*, which produced 5-hydroxytryptophan subsequently converted to serotonin, promoting colorectal tumor progression. This effect is mechanistically blocked by inhibiting the 5-HTP-to-serotonin conversion step (Zhu et al., 2026). This finding is specific to intestinal rather than brain tumors; however, it independently demonstrates that aspartame can drive tumor-promoting effects through a defined microbiota–metabolite mechanism. Additionally, the involvement of the tryptophan–serotonin pathway connects directly to the tryptophan–kynurenine axis discussed in Section 3.3.3.

#### 3.2.6 Hypothesized mechanistic sequence linking dietary additives, gut dysbiosis, and glioblastoma

No other additive in this review has evidence connecting it to gut dysbiosis, epithelial barrier compromise, and glioblastoma-specific epigenetic reprogramming within a single chain, further reinforced by directionally opposite evidence from an independent dietary intervention (restriction). However, the chain is not without gaps; the core glioblastoma finding did not observe a tumor-growth effect, human validation is based on correlation (TCGA/CGGA expression data), and the gut-microbiome and glioblastoma-epigenome studies were conducted in different model systems rather than as a single continuous experiment. Despite this, among all additives evaluated, aspartame currently provides the most complete hypothesized mechanistic sequence linking dietary exposure to gut dysbiosis, epithelial dysfunction, epigenetic reprogramming, and glioblastoma-associated signaling. The individual metabolite-level links underlying this continuum are summarized in **Table 4**, while **Figure 2** summarizes the proposed mechanistic sequence linking dietary aspartame exposure to glioblastoma through gut microbiota dysbiosis and m^6^A-mediated epigenetic reprogramming.

**Table 4:** Microbial products, metabolites, and host mediators linking gut dysbiosis to glioblastoma.

Molecular class/mediator category	Source taxa/origin	Effect on gut	Effect on brain	GBM pathway/relevance	References
Tryptophan-pathway derivative					
Kynurenic acid	Produced via tryptophan metabolism by *Lactobacillus* sp., *Bifidobacterium* sp.	↓ Following sucralose-induced dysbiosis; protective anti-inflammatory tryptophan metabolite; loss shifts the kynurenine pathway toward the neurotoxic arm	↓ Cancer cell adhesion to brain vasculature; neuroprotective; loss removes a protective barrier at the BBB level	Controls metastasis initiation; depletion permits cancer cell adhesion to cerebral vasculature	(Bian et al., 2017a; Massara et al., 2025)
Host tight-junction proteins					
Claudin-1,3,4	Expression maintained by SCFA-producing bacteria (*Bifidobacterium* sp., *Lactobacillus* sp., *Faecalibacterium* sp.); depleted by food additives across multiple categories	↓ By sucralose/aspartame/saccharin via T1R3 receptor activation and ROS; ↑ intestinal permeability; ↓ occludin and ZO-1 co-downregulated	Parallel loss of claudin-5 at BBB (propionate depletion removes FFAR2-mediated maintenance); ↑ BBB permeability	Two-barrier failure enables translocation of LPS, kynurenine, and quinolinic acid to brain parenchyma → TGF-β/IL-10 immunosuppression	(Li et al., 2020; Shil et al., 2020; Guo et al., 2021; Chenghan et al., 2025)
Microbial structural product/endotoxin					
LPS (lipopolysaccharide)	↑ From expanded Gram-negative bacteria: Enterobacteriaceae, Proteobacteria, *E. coli* (driven by sweeteners, preservatives)	↑ LPS biosynthesis pathway genes (saccharin); ↑ systemic endotoxemia via compromised intestinal barrier	Activates TLR4 → NF-κB → ↑ TNF-α, IL-6, IL-1β, COX-2; drives microglial activation and chronic neuroinflammation	TLR4/NF-κB signaling may promote angiogenesis, inflammatory signaling, and immune evasion; COX-2/PGE signaling may contribute to a pro-tumor inflammatory microenvironment. Direct evidence in GBM remains limited and should be considered mechanistic/emerging rather than established.	(Bian et al., 2017b; Gong et al., 2026)
Microbial metabolite					
Bile acids	Secondary bile acid conversion by *Bifidobacterium* sp. microbial dysbiosis can alter the balance and composition of primary and secondary bile acids	Altered primary/secondary ratio (↑ CA, CDCA, TCDCA); act as signaling molecules; affect immune cell differentiation	Circulating bile acids can influence the gut–brain axis; some bile acids can reach the CNS and modulate neuroinflammatory and BBB-related signaling through receptors including FXR and TGR5	Altered bile-acid signaling may influence GBM-relevant immune and metabolic pathways. Emerging evidence implicates bile-salt synthesis/signaling in GBM immune privilege and tumor biology, but a direct causal link between gut dysbiosis-induced bile-acid changes and GBM progression remains unestablished.	(Bian et al., 2017b; Sharpe et al., 2023; Green et al., 2024; Li et al., 2024; Beales et al., 2026; Gong et al., 2026)
Host mediator/neurotransmitter					
Serotonin	↑ Via Red 40-induced colonic serotonin production (microbiota-dependent and microbiota-independent); ↑ long-term by silver nanoparticles	↑ Pro-inflammatory cytokines (IL-1β, IL-6, TNF-α); ↓ ZO-1 tight junctions; increased colitis susceptibility	Neuromodulatory; altered serotonin levels affect neural signaling and neuroinflammation; persistent disruption despite microbiota recovery (silver NPs)	Modulates glioma cell proliferation and invasion; serotonin receptor signaling implicated in GBM cell biology	(Dono et al., 2020; Wang et al., 2023)

↑, increased or elevated; ↓, decreased or reduced; →, leads to or signals to (proposed mechanistic direction; does not imply established causality in glioblastoma).

**Figure 2:**
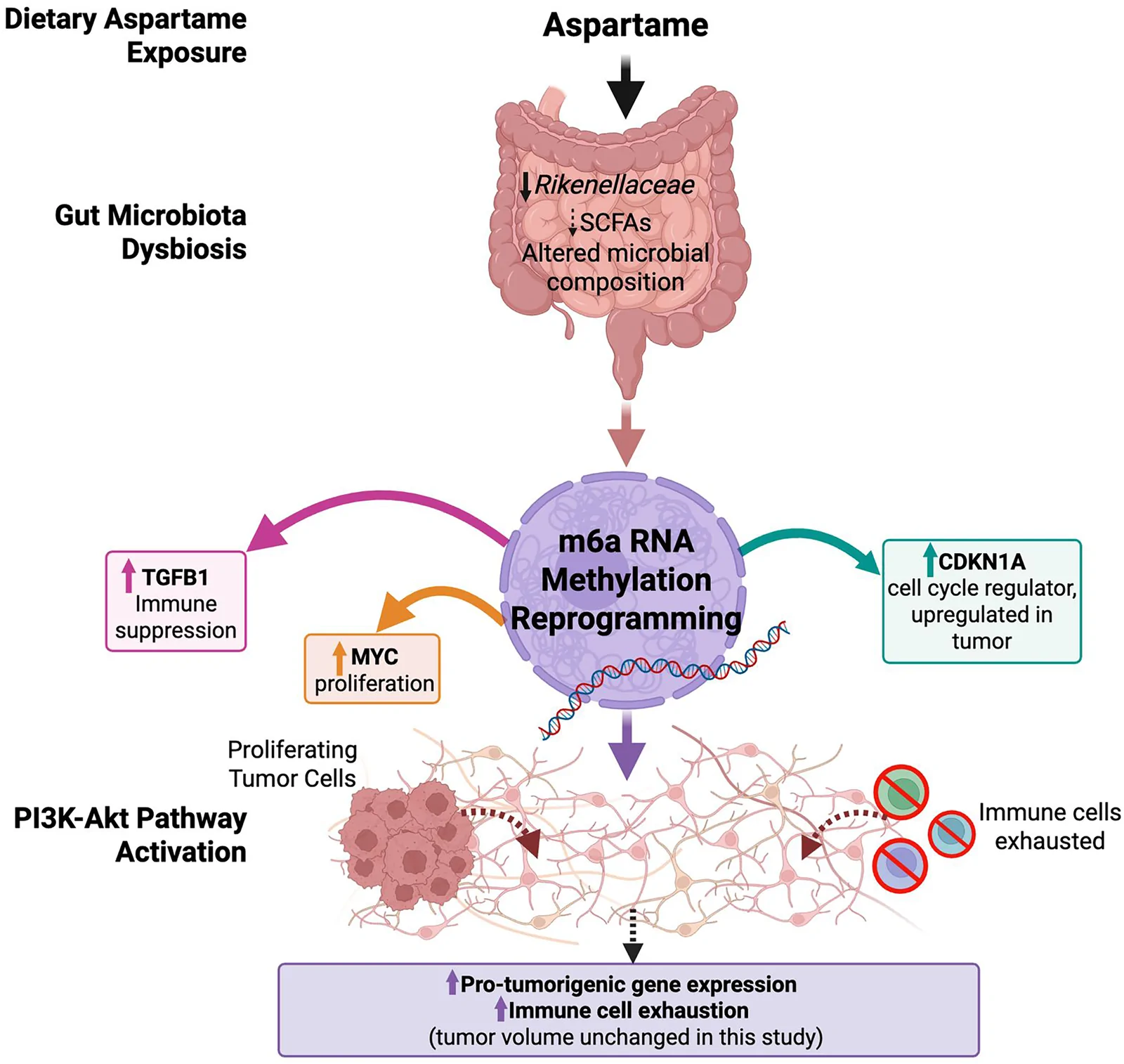
Proposed mechanistic pathway by which dietary aspartame exposure promotes glioblastoma (GBM) progression via gut microbiota dysbiosis and m^6^A RNA methylation reprogramming: Dietary aspartame ingestion alters the gut microbiota composition, characterized by a reduction in Rikenellaceae abundance, decreased SCFA production, and broader shifts in microbial community structure. These dysbiotic changes drive reprogramming of m^6^A RNA methylation, a key post-transcriptional regulatory mechanism. m^6^A reprogramming subsequently activates multiple pathways: upregulation of *TGFB1* (immune suppression); increased *MYC* expression (tumor cell proliferation); and upregulation of *CDKN1A*; leading to PI3K–Akt pathway activation. These molecular alterations are consistent with a pro-tumorigenic microenvironment characterized by increased proliferative and immunosuppressive signaling; however, aspartame exposure did not significantly alter tumor growth in the source study, indicating these effects reflect epigenetic and molecular reprogramming independent of measurable changes in tumor volume. Arrows indicate directional regulatory relationships; ↑ denotes upregulation or increase; ↓ denotes downregulation or decrease. SCFAs, short-chain fatty acids; *TGFB1*, transforming growth factor beta 1; *CDKN1A*, cyclin-dependent kinase inhibitor 1A; GBM, glioblastoma multiforme. Solid arrows = experimentally demonstrated in the cited study; dashed arrows = proposed mechanistic link. Created in BioRender. Busselberg, D. (2026). https://BioRender.com/tvw4j5m.

### 3.3 Metabolite-mediated signaling across the gut–brain axis

This section discusses the principal metabolite classes through which additive-induced dysbiotic changes communicate with the central nervous system, synthesizing findings from both metabolomic datasets. The preceding sections establish that food additives consistently alter microbial ecology. The critical question therefore becomes how these intestinal alterations influence distal organs such as the brain. Current evidence indicates that this communication occurs primarily through microbial metabolites capable of regulating immune signaling, barrier integrity, neurotransmission, and epigenetic pathways. Because direct evidence linking food additive-induced metabolite changes to GBM remains limited, this section draws on both GBM-specific and non-GBM studies. The evidence source for each finding is indicated in the text to enable readers to assess the directness of each claim.

#### 3.3.1 Short-chain fatty acids

Among the metabolite classes disrupted by additive-induced dysbiosis, SCFAs were the most consistently reported and well-characterized metabolite class. A clinical cohort study of 70 breast cancer patients demonstrated that chemotherapy depleted fecal SCFAs in a pattern paralleling food additive-induced depletion (clinical, human breast cancer cohort) (Beales et al., 2026). Butyrate was shown to cross the BBB and exert anti-inflammatory effects by reducing TNF-α and IL-1β while increasing IL-4 and IL-10, promoting microglial M1 to M2 polarization (preclinical, rat traumatic brain injury model) (Wei et al., 2026). Sodium butyrate functioned as an HDAC inhibitor, enhancing histone acetylation and upregulating PD-L1 via PI3K/AKT, improving anti-PD-1 immunotherapy efficacy in glioma (preclinical, glioma mouse/human model) (Li et al., 2025). An *in vitro*/*in silico* study elucidated the molecular mechanism: butyrate binds the inositol-tetraphosphate site of the HDAC3–NCoR2 complex, allosterically inhibiting HDAC3 activity and suppressing *EGFR* expression (*in vivo*, glioblastoma) (Ashwini et al., 2025). The studies drawn on here are not equivalent. They comprise a clinical breast cancer chemotherapy cohort, a rat traumatic brain injury model, an orthotopic GL261 glioma model treated with intraperitoneal sodium butyrate at 1.2 g/kg daily for 4 weeks, an *in vitro* and *in silico* mechanistic study, and an orthotopic glioma probiotic gavage study. Only the last two administered an agent to a tumor-bearing animal. In the butyrate and anti-PD-1 study, butyrate raised PD-L1, a pro-tumor marker, and helped only when combined with checkpoint blockade, so SCFA supplementation is not straightforwardly protective (see **Supplementary Table** for details).

Probiotic supplementation with *Bifidobacterium lactis* and *L. plantarum* (the taxa most consistently depleted by food additives) suppressed PI3K/AKT signaling and decreased tumor volume in a glioma mouse model (preclinical, glioma mouse model) (Wang et al., 2022). Propionate uniquely restored BBB tight junction integrity via FFAR2, in a mechanism distinct from butyrate and acetate (preclinical, mouse GBM model) (Chenghan et al., 2025).

#### 3.3.2 LPS and systemic endotoxemia

A Mendelian randomization (MR) study in meningioma supported a potential causal relationship between arachidonic acid-mediated COX-2/PGE2 and LPS-driven TLR4/NF-κB activation and brain tumor inflammation and immune evasion; while this evidence derives from a different brain tumor type rather than GBM directly, it supports biological plausibility for LPS/TLR4-mediated neuroinflammatory mechanisms in brain tumors more broadly (Mendelian randomization, meningioma) (Gong et al., 2026). Three independent clinical studies corroborated this: a case–control study of 40 patients identified elevated Proteobacteria and *E. coli* with reduced *Faecalibacterium* sp. (clinical, human glioblastoma) (Ishaq et al., 2024), a pilot of 100 participants found *Fusobacterium* overrepresentation in brain tumor patients (Jiang et al., 2022) (clinical, mixed benign/malignant brain tumors); and 80 participants confirmed reduced microbial richness in GBM (clinical, human glioblastoma) (Jiang et al., 2024). An MR study supported a potential causal relationship between *Eubacterium brachy* and increased GBM risk, while identifying *Prevotella7* as potentially protective (Mendelian randomization, glioblastoma) (Zeng et al., 2023). **Figure 3** illustrates the dual inflammatory mechanisms by which gut dysbiosis promotes a glioblastoma-supportive neuroinflammatory microenvironment through SCFA depletion and LPS-mediated signaling.

**Figure 3:**
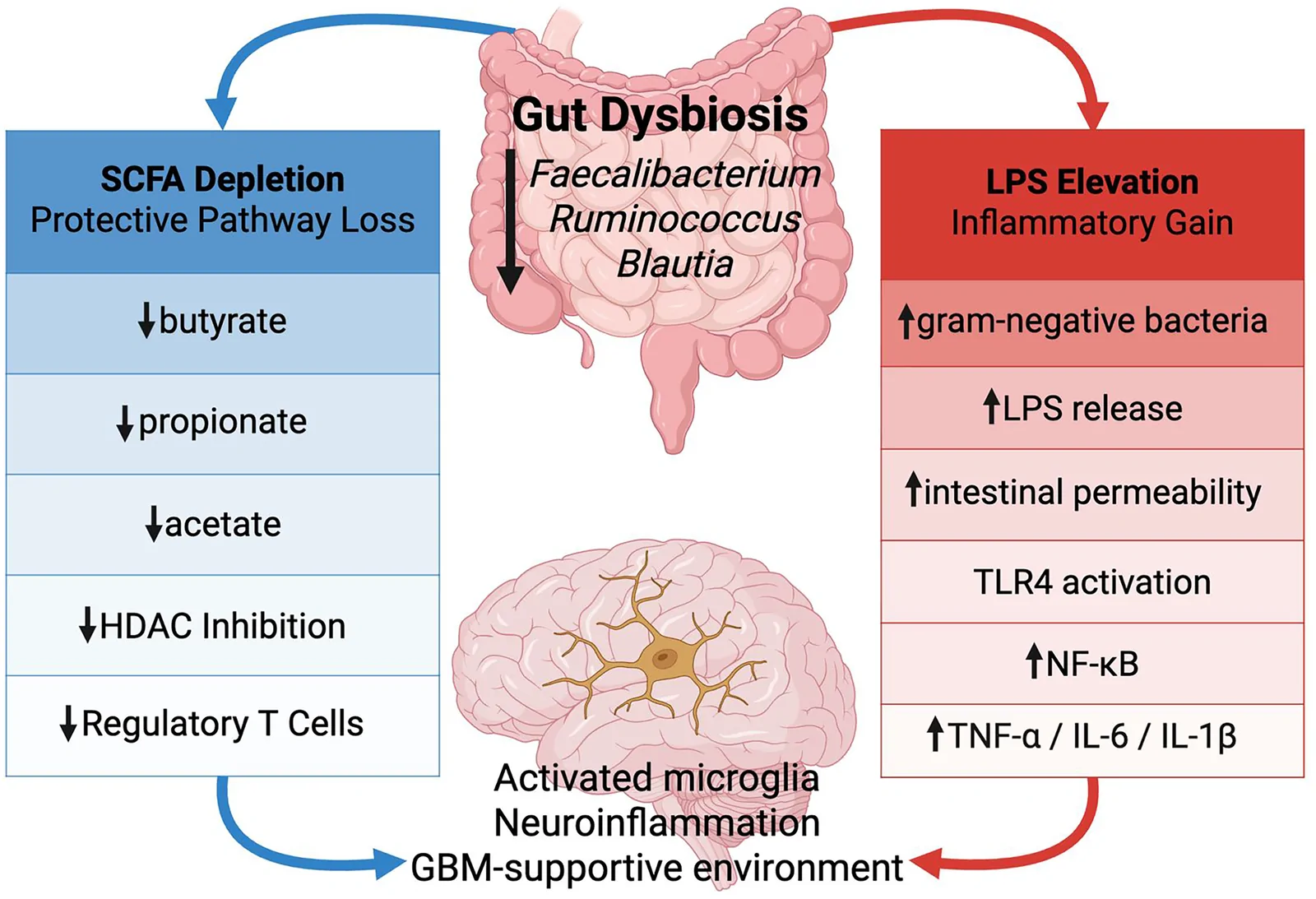
Dual mechanisms by which gut dysbiosis promotes a GBM-supportive neuroinflammatory environment via SCFA depletion and LPS elevation: Gut dysbiosis, characterized by reduced abundance of SCFA-producing commensal bacteria *Faecalibacterium* sp., *Ruminococcus* sp., and *Blautia* sp., drives two convergent pathological pathways that collectively activate microglia and establish a neuroinflammatory, GBM-supportive environment. On the left, depletion of SCFA-producing bacteria results in decreased levels of butyrate, propionate, and acetate, leading to loss of histone deacetylase (HDAC) inhibition and reduced regulatory T-cell populations. On the right, a concurrent shift toward Gram-negative bacterial dominance elevates lipopolysaccharide (LPS) release and increases intestinal permeability. This activates Toll-like receptor 4 (TLR4) signaling, upregulates NF-κB, and drives the production of pro-inflammatory cytokines including TNF-α, IL-6, and IL-1β. Together, these two arms converge to activate microglia and sustain chronic neuroinflammation, creating a tumor microenvironment permissive to GBM progression. ↑ denotes upregulation or increase; ↓ denotes downregulation or decrease. SCFAs, short-chain fatty acids; LPS, lipopolysaccharide; HDAC, histone deacetylase; TLR4, Toll-like receptor 4; NF-κB, nuclear factor kappa B; TNF-α, tumor necrosis factor alpha; IL, interleukin; GBM, glioblastoma multiforme. Created in BioRender. Busselberg, D. (2026). https://BioRender.com/tvw4j5m.

#### 3.3.3 Tryptophan–kynurenine pathway and aryl hydrocarbon receptor signaling

The tryptophan–kynurenine pathway represents another route through which dysbiosis-derived metabolites reach the brain. An MR study found that microbiota-derived metabolites activating AHR, IDH-mediated epigenetic pathways, and microglial dysfunction were associated with a potential causal relationship to GBM risk (Yan et al., 2024). Kynurenic acid (KYNA), whose levels decreased following sucralose exposure (Bian et al., 2017a), was found to inhibit cancer cell adhesion to brain vasculature, controlling metastasis initiation (Massara et al., 2025). Additional MR analyses implicated xenobiotic stress and neurosteroid (DHEAS) signaling as mediators (Chen et al., 2024).

#### 3.3.4 Bile acid disruption

Saccharin altered bile acid biosynthesis pathways (Bian et al., 2017b). The clinical SCFA cohort study documented elevated primary bile acids (CA, CDCA, TCDCA) (Beales et al., 2026). Routine GBM medications similarly altered bile acid metabolism alongside SCFA and tryptophan pathways (Green et al., 2024).

#### 3.3.5 Blood–brain barrier disruption

The intestinal barrier disruption documented across sweeteners (Shil et al., 2020; Guo et al., 2021; Kwon et al., 2022) has a direct anatomical parallel at the BBB. Propionate maintained BBB tight junction protein expression via FFAR2, whereas acetate and butyrate could not reverse antibiotic-induced BBB permeability (Chenghan et al., 2025). **Figure 4** depicts how gut dysbiosis compromises the intestinal and blood–brain barriers, facilitating neuroinflammation, immune evasion, and glioblastoma progression.

**Figure 4:**
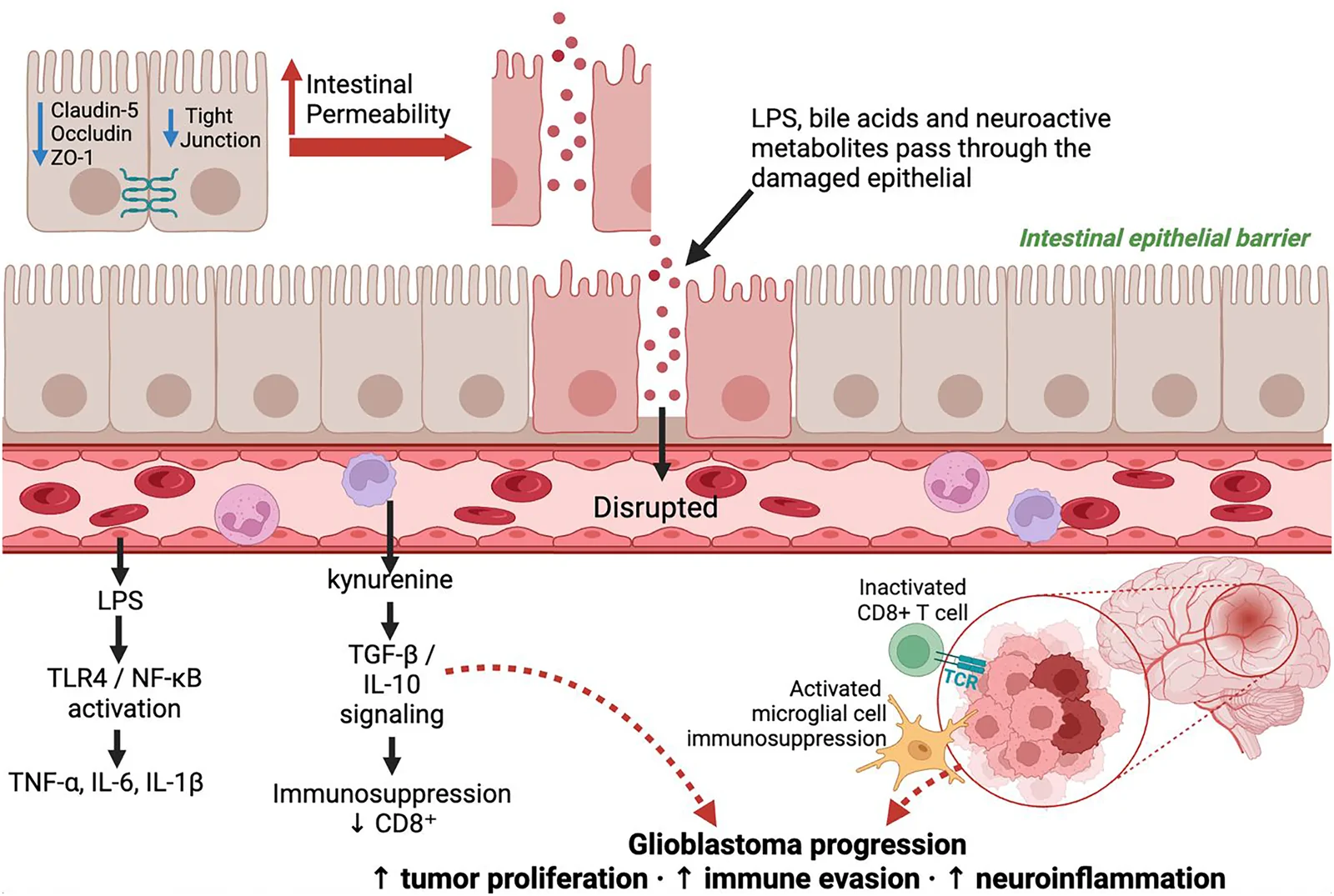
Gut dysbiosis-driven blood–brain barrier disruption and glioblastoma progression: Gut dysbiosis depletes SCFA-producing commensal populations and expands LPS-associated and Gram-negative communities, compromising tight junction proteins (claudin-5, occludin) and increasing intestinal permeability. This permits translocation of LPS, pro-inflammatory cytokines, and neuroactive metabolites (kynurenine, quinolinic acid) into systemic circulation, where they subsequently disrupt the blood–brain barrier. Within the brain compartment, LPS activates TLR4/NF-κB signaling, driving microglial neuroinflammation, while kynurenine promotes TGF-β/IL-10-mediated immunosuppression, collectively increasing tumor invasiveness, neuroinflammation, and immune evasion in glioblastoma. This figure synthesizes evidence from GBM-specific studies alongside findings from glioma, meningioma, and non-tumor models (e.g., traumatic brain injury), reflecting the current state of the literature; however, not every step has been directly demonstrated in glioblastoma. See main text for source-specific evidence annotations. Created in BioRender. Busselberg, D. (2026). https://BioRender.com/tvw4j5m.

### 3.4 Glioblastoma progression via the gut–brain axis

#### 3.4.1 Immune checkpoint modulation and the tumor microenvironment

The glioblastoma tumor microenvironment (GBM TME) is a heterogeneous and dynamic system composed of tumor cells and diverse cellular and non-cellular components. Cellular components include endothelial cells, neurons, astrocytes, oligodendrocytes, resident immune cells such as microglia, tumor-associated macrophages, and tumor-infiltrating lymphocytes, while non-cellular components include signaling molecules, exosomes, extracellular matrix components, and extracellular matrix-remodeling enzymes. These components interact with glioma cells through direct cell–cell interactions and soluble mediators, influencing tumor survival, progression, and therapeutic response (Sharma et al., 2023).

Antibiotic-induced gut dysbiosis in murine glioma models (GL261) reduced *Foxp3* expression in the brain, resulting in loss of regulatory T-cell-mediated immune control and accelerated glioma progression in preclinical models; FMT reversed this effect (Fan et al., 2022). MR analyses supported a potential causal relationship between gut-derived cytokines (IL-10, TGF-β, IL-23) and upregulation of PD-L1 and IDO, promoting immune suppression, while Peptostreptococcaceae and *Coprobacter* sp. were identified as potentially protective (Cui et al., 2024). Probiotic *Bifidobacterium* supplementation reversed this immunosuppressive state through downregulation of PD-1/PD-L1 and NF-κB, reducing tumor volume (Fan et al., 2024). Oncolytic virotherapy enhanced CD8^+^ T-cell immunity by restoring *Bifidobacterium* and *Akkermansia* sp., producing lactate converted to butyrate (preclinical, mouse GBM model) (Meléndez-Vázquez et al., 2024).

These findings carry particular significance: the taxa whose restoration enhances antitumor immunity (*Bifidobacterium* sp., *Akkermansia* sp., *Lactobacillus* sp.) are precisely those depleted by the recurrent, though non-universal, dysbiotic pattern documented in Section 3.1.

#### 3.4.2 Epigenetic and AHR-mediated pathways

Beyond immune modulation, Mendelian randomization analyses have provided genetic evidence for causal relationships between gut microbiota and GBM risk. Multiple MR studies supported potential causal relationships between specific gut taxa and GBM risk, with Bacteroidaceae exhibiting OR = 12.0 and Ruminococcaceae OR = 0.094 (protective) (Ju et al., 2024). Additional MR analyses confirmed 211 taxa were screened, of which 9 demonstrated nominally significant associations with GBM by IVW analysis; however, only the Ruminococcaceae family (OR = 0.04, *p*FDR = 0.003) retained significance after Benjamini–Hochberg correction (Wang et al., 2024) and AHR activation, IDH modulation, and *Foxp3*/Treg dysregulation as potential causal mediators (Yan et al., 2024). A multitumor-type MR study with machine learning identified 63 glioma-related microbe-related genes expressed in brain tissue (Wu et al., 2025). The aspartame–m^6^A–PI3K/AKT axis (Meng et al., 2025) converges with these MR-identified epigenetic axes, suggesting that food additive-driven dysbiosis may simultaneously activate multiple epigenetic programs.

## 4 Innovations, challenges, limitations, and future perspectives

While the preceding sections have synthesized the mechanistic evidence linking food additive-induced gut dysbiosis to glioblastoma progression via the gut–brain axis, it is equally important to position this work within the broader landscape, candidly assess the limitations, and articulate a forward-looking research agenda.

### 4.1 Innovations

This review makes several contributions that distinguish it from prior work. Previous reviews have examined food additives to microbiome and microbiome to cancer domains in isolation; the present work bridges this gap by constructing a unified mechanistic framework tracing the complete continuum from food additive ingestion through gut dysbiosis, metabolite disruption, BBB compromise, and activation of oncogenic pathways within the GBM tumor microenvironment. To our knowledge, no prior review has linked these domains end-to-end. Existing reviews cluster around one of three narrower foci: broad microbiome–cancer mechanisms without a food-additive or organ-specific focus (Constantin et al., 2024); food additive–microbiome interactions confined to gastrointestinal and metabolic outcomes without extension to neuro-oncology (Alum et al., 2025; Seto et al., 2025); or general gut–brain axis physiology without a cancer or dietary-additive view (Sasso et al., 2023). The present review differs from each by tracing a single, additive-specific causal chain from ingestion, through dysbiosis and metabolite disruption, to glioblastoma-relevant molecular endpoints, rather than treating them as separate literature.

A second innovation is the recurrent, though non-universal, dysbiotic pattern across chemically diverse additive categories. Rather than treating each additive as an isolated perturbation, this review demonstrates that sweeteners, preservatives, colorants, nanoparticles, and emulsifiers converge on a consistent pattern (depletion of *Bifidobacterium*, *Lactobacillus*, *Faecalibacterium*, and *Akkermansia* alongside expansion of Proteobacteria), transforming the inquiry from an additive-specific problem into a generalizable dietary pattern question. Recognizing a recurrent, though non-universal, dysbiotic pattern matters for two practical reasons. First, a convergent signature is a more yielding biomarker target than dozens of additive-specific ones; a panel built around depleted *Bifidobacterium*/*Lactobacillus*/*Faecalibacterium*, reduced SCFA output, and elevated LPS could in principle flag additive-driven dysbiosis regardless of which specific compound(s) a person consumes, which is more realistic given that real-world exposure is most likely a mixture (Gerasimidis et al., 2020; Liu et al., 2022; Li et al., 2024). Second, it supports interventions rather than requiring compound-by-compound regulatory or clinical responses; microbiome-targeted strategies (probiotics restoring depleted taxa, dietary fiber to support SCFA production) could plausibly address the downstream consequences of many additives at once.

A third of this review is that the metabolite-centered synthesis organizes gut–brain axis evidence by functional metabolite classes (SCFAs, LPS, tryptophan-kynurenine derivatives, and bile acids) rather than bacterial taxonomy, mirroring the biological logic of the axis and providing a more usable framework for therapeutic intervention. Finally, the aspartame proof-of-concept model traces a single additive from T1R3-mediated barrier disruption through Rikenellaceae depletion to m^6^A RNA methylation reprogramming and PI3K-AKT activation, juxtaposed with the mirror reversal achieved by intermittent fasting through the same m^6^A–TGF-β mechanistic axis. This establishes a falsifiable model that future studies can test directly. One option is a longitudinal design, using repeated fecal microbiome sampling paired with tumor m^6^A profiling in the same animals over time. Another is patient-derived GBM organoids grown with specific, known bacterial communities, with and without aspartame, to separate the microbiome’s role from any direct effect of the compound on the tumor. Validation could also happen at the receptor level: computer simulations of aspartame binding to the human sweet-taste receptor T1R2–T1R3 have already mapped the exact amino acids the receptor uses to recognize and bind aspartame (Tong et al., 2026). This fits with lab evidence that a related sweetener, neotame, damages the gut barrier both directly through this same receptor and indirectly by changing gut bacteria (Shil et al., 2024). Together, this suggests aspartame’s barrier and microbiome effects could be experimentally separated by testing receptor activation in cells with the receptor gene knocked out.

### 4.2 Challenges

The most fundamental challenge is the predominance of animal studies. Mouse gut microbiomes share only approximately 10% of species-level taxa with humans, and they cannot fully stand in for the complexity of a real human microbiome. This shows up directly in the GBM literature, where researchers have used humanized microbiome mouse models of glioma, mice colonized with human donor gut bacteria, specifically because standard mouse models cannot capture how human gut communities affect the response to immunotherapy in glioma (Dees et al., 2021). Even this approach only partly fixes the problem, since one round of humanization cannot reproduce how a real human gut community keeps changing in response to diet. A second challenge is heterogeneous additive dosing. Many preclinical studies administer concentrations exceeding typical human dietary intake, making dose–response extrapolation difficult. The few studies employing doses within acceptable daily intake ranges are notable precisely because they are exceptional. Third, diverse sequencing and analytical methods used by different studies introduce comparability challenges, as some use 16S rRNA sequencing, which reads only one gene to identify bacteria, whereas others use shotgun metagenomics, which sequences more of the genome and can detect more species overall (Chitcharoen et al., 2025). The absence of standardized metabolomics pipelines is particularly problematic for the metabolite-centered framework proposed here, as SCFA, LPS, and tryptophan measurements vary substantially across platforms, and even carefully controlled comparisons between labs using the same standardized metabolomics kit still find real variability, with some metabolites showing close to 20% variation between labs (Siskos et al., 2017). Finally, the near-complete absence of longitudinal human studies represents a structural challenge. The available human evidence is predominantly cross-sectional, with microbiome composition and clinical outcome assessed at a single point in time. Such designs can establish association but cannot resolve temporal sequence, leaving open the question of whether observed dysbiosis precedes disease onset or arises as a consequence of it. MR studies address this limitation through a different mechanism, using genetic variants as proxies for exposure to enable causal inference independent of temporal confounding. However, MR is constrained by its reliance on fixed, germline genetic variation as an instrument; it cannot capture exposures that vary continuously across the lifespan, such as an individual’s cumulative or fluctuating dietary additive intake, and therefore cannot substitute for direct longitudinal measurement of environmental exposure (‘Mendelian Randomization’: Can Genetic Epidemiology Contribute to Understanding Environmental Determinants of Disease?1 | International Journal of Epidemiology | Oxford Academic). Addressing this gap will require prospective cohort studies that follow participants over extended periods, with concurrent tracking of additive intake and metabolic/neurological outcomes. The value of such cohorts would be substantially enhanced by adopting validated dietary assessment instruments alongside harmonized microbiome sampling and analytical protocols across research groups, which would improve cross-study comparability and reduce the methodological heterogeneity that currently limits synthesis across the existing evidence base (Diacova et al., 2025).

### 4.3 Limitations

This is a narrative review rather than a systematic review or meta-analysis and does not employ quantitative synthesis or statistical pooling of effect sizes. While this approach is well suited to integrating diverse forms of evidence, it is inherently susceptible to selection bias. To minimize this risk, literature was retrieved from four databases (PubMed, Scopus, ScienceDirect, and Google Scholar) using predefined search strategies for the two evidence domains (Section 2). Duplicate records were removed using Zotero reference management software, and studies were screened against predefined inclusion criteria (peer-reviewed original research, English language, 2015–2026) before full-text review. Although these measures improve the transparency and consistency of study selection, the present review should be interpreted as an integrative conceptual synthesis rather than a definitive quantitative assessment. Furthermore, publication bias cannot be excluded, as studies reporting significant associations are generally more likely to be published than those reporting null or protective findings. The paradoxical SCFA-increasing effects of xylitol observed across three studies illustrate that the prevailing narrative of additive-mediated harm is not universal and highlight the importance of considering contradictory evidence.

While this review proposes a hypothesized mechanistic sequence from food additive exposure to GBM progression, the current evidence does not establish causality for this complete chain. The additive-to-dysbiosis and dysbiosis-to-GBM links are individually supported, but the end-to-end pathway has not been demonstrated in any single study, in any species. The framework here is a biologically plausible hypothesis backed by strong mechanistic evidence; however, it is not a proven causal relationship. It should be read as a hypothesis-generating, structured starting point for the prospective experimental and clinical studies described below, not a claim that causation has already been shown. The clinical evidence is limited to cross-sectional case–control studies with small sample sizes (*n* = 25–100), none of which were prospective or controlled for dietary additive intake. MR studies try to get around this by using genetic variants as stand-ins for an exposure, which does provide causal inference at the level of genetically predicted microbiome variation but cannot directly speak to changeable environmental dietary exposures (‘Mendelian Randomization’: Can Genetic Epidemiology Contribute to Understanding Environmental Determinants of Disease?1 | International Journal of Epidemiology | Oxford Academic).

### 4.4 Future perspectives

Future research should focus on large, prospective human cohort studies that track dietary additive intake alongside changes in the gut microbiome and long-term glioblastoma outcomes, a study design that does not yet exist for this specific research question. Existing cohorts, such as the UK Biobank (UK Biobank: An Open Access Resource for Identifying the Causes of a Wide Range of Complex Diseases of Middle and Old Age | PLOS Medicine), the Nurses’ Health Study (Origin, Methods, and Evolution of the Three Nurses’ Health Studies | AJPH | Vol. 106 Issue 9), and the European Prospective Investigation into Cancer and Nutrition (EPIC) (European Prospective Investigation Into Cancer and Nutrition (EPIC): Study Populations and Data Collection | Public Health Nutrition | Cambridge Core), could potentially be reanalyzed to investigate additive consumption and compare individuals who develop GBM with matched controls. Future cohort studies should use validated diet-tracking tools that capture specific additive-containing products, not just broad food groups, as a general food frequency questionnaire is unlikely to be a precise tool to estimate quantities of additive intake (Trakman et al., 2020; Hall and Trakman, 2025). Repeated microbiome sampling would allow differentiation between short-term dysbiosis and lasting alterations in the environment. Furthermore, the human gut microbiome has been shown to remain reasonably stable over time in healthy adults; therefore, a single sample may provide an inaccurate description (Faith et al., 2013). Multi-omics approaches would also strengthen this evidence base. Beyond shotgun metagenomics, metabolomics, single-cell RNA sequencing, and spatial transcriptomics, this should extend to spatial metabolomics and broader host–microbiome multi-omics integration, which allow researchers to localize where a given metabolite or gene expression change is occurring rather than averaging across an entire sample, a distinction that matters considerably for gut–brain axis research, where the anatomical compartment involved (gut, tumor, or brain tissue) is mechanistically important (Full Article: Multi-Omic Approaches for Host-Microbiome Data Integration). Clinical trials should also evaluate microbiome-targeted interventions, such as probiotics, fecal microbiota transplantation, and dietary modification, alongside standard GBM treatments to determine their therapeutic potential. In addition, artificial intelligence and machine learning could integrate dietary, microbiome, metabolite, and clinical data to identify high-risk individuals and support personalized prevention strategies. Such models risk functioning as uninterpretable “black boxes,” therefore, incorporating explainable AI (XAI) methods will be important for clinical adoption, as these approaches make a model’s reasoning more transparent and allow clinicians to use the tool to evaluate predictions rather than accept them uncritically (Caterson et al., 2024). Finally, future studies should investigate the combined effects of multiple food additives rather than individual compounds, since real-world dietary exposure occurs as mixtures rather than single substances, a pattern already documented in a large cohort of French adults exposed to specific, identifiable additive combinations in their everyday diets (Exposure to Food Additive Mixtures in 106,000 French Adults From the Nutrinet-Santé Cohort | Scientific Reports). Characterizing these mixture effects, rather than continuing to study additives in isolation, would provide considerably stronger evidence to inform future dietary policy and regulatory decision-making.

## 5 Conclusion

This review proposes a mechanistic framework linking dietary food additive exposure to glioblastoma through the gut microbiome. Five chemically distinct additive categories—artificial sweeteners, food colorants, preservatives, nanoparticles, and emulsifiers—were shown to converge on a recurrent, though non-universal, dysbiotic pattern characterized by depletion of SCFA-producing taxa, expansion of pro-inflammatory and Gram-negative communities, and disruption of the tryptophan–kynurenine and lipopolysaccharide pathways. This convergence, evident despite the additives’ unrelated chemical structures and intended functions, suggests that gut dysbiosis represents a shared downstream response rather than an additive-specific idiosyncrasy, though, as Section 3.1.6 notes, this signature is not universal, and boundary conditions (regulatory-dose human trials, direct protein-binding routes) temper any claim of a single, deterministic pathway. Aspartame provided the clearest exemplar of this pathway in action, with evidence spanning microbiome disruption, epithelial barrier compromise, and m^6^A-mediated upregulation of *TGFB1*, *MYC*, and *CDKN1A* in glioblastoma tissue, a continuum not yet demonstrated for any other single additive. The subsequent report that intermittent fasting suppresses the same gut-microbiota–m^6^A–TGF-β axis in a *Tp53*-driven glioma model, acting in the opposite direction to aspartame’s effect, offers the strongest available bidirectional support for this mechanism, though the two studies implicate different TGF-β family members and were conducted as independent experiments rather than a single continuous study. Downstream of this shared dysbiotic signature, four functional metabolite classes, SCFAs, LPS, tryptophan–kynurenine derivatives, and bile acids, were each independently linked to blood–brain barrier compromise, neuroinflammation, and immune checkpoint modulation within the glioblastoma tumor microenvironment, with the taxa most consistently depleted by food additives (*Bifidobacterium* sp., *Lactobacillus* sp., *Akkermansia* sp.) matching those whose experimental restoration enhances antitumor immunity in glioma models. Taken together, these findings can advance the field by reframing food additive exposure from an isolated, additive-by-additive toxicological question into a systems-level, microbiome-mediated one, with practical relevance for both prevention and treatment, as the same depleted taxa that plausibly initiate dysbiosis are also targets whose restoration has shown therapeutic promise in preclinical glioma models. By organizing gut–brain axis evidence around functional metabolite classes rather than bacterial taxonomy, this review offers a more direct route to testable biomarkers and intervention than a purely taxonomic account would. Several priorities should guide the next phase of research. Prospective human cohorts that pair validated dietary-additive exposure assessment with longitudinal microbiome sampling and glioblastoma outcomes are most needed, given that current human evidence remains cross-sectional or genetically inferred. Multi-omics approaches could clarify which of the mechanistic links identified here operate in human tumors rather than murine models alone. Because real-world dietary exposure involves simultaneous consumption of multiple additives rather than isolated compounds, studies of additive mixtures, of which only one currently exists in this evidence base, represent a particularly urgent gap. Finally, the aspartame–m^6^A–*TGFB1* axis proposed here is a model that future studies could directly test, offering a concrete starting point for validation of mechanisms. The available evidence suggests that dietary food additives should no longer be viewed solely as technological ingredients but as biologically active modifiers of the gut microbiome capable of reshaping metabolite signaling, intestinal barrier integrity, and neuroimmune communication. While direct clinical evidence linking additive exposure to glioblastoma remains limited, the converging mechanistic data presented in this review provide a rationale for considering dietary additives as hypothesized environmental contributors to glioblastoma pathogenesis that are potentially modifiable. Confirming this role will require prospective epidemiological studies and direct mechanistic validation in human tissue; at present, this framework should be read as hypothesis-generating rather than an established causal model, with microbiome-informed prevention and therapy representing emerging, not yet validated, targets.

## Supplementary material

Supplementary Table 1 Experimental model, exposure conditions (substance, dose, route, duration), analytical method, and null or conflicting findings for every primary study included in the synthesis.

## Glossary

ADI: acceptable daily intake
AHR: aryl hydrocarbon receptor
ANSA-Na: 1-amino-2-naphthol-6-sulfonate sodium salt
BBB: blood–brain barrier
BCFA: branched-chain fatty acid
BHA: butylated hydroxyanisole
CA: cholic acid
CD4/CD8: cluster of differentiation 4/8
CDCA: chenodeoxycholic acid
*CDKN1A*: cyclin-dependent kinase inhibitor 1A
CGGA: Chinese Glioma Genome Atlas
CMC: carboxymethylcellulose
CNS: central nervous system
COX-2: cyclooxygenase-2
DHEAS: dehydroepiandrosterone sulphate
DSS: dextran sulfate sodium
EDTA: ethylenediaminetetraacetic acid
EFSA: European Food Safety Authority
*EGFR*: epidermal growth factor receptor
EPIC: European Prospective Investigation into Cancer and Nutrition
FDA: Food and Drug Administration
FFAR2: free fatty acid receptor 2
FMT: fecal microbiota transplantation
GBM: glioblastoma
GRAS: Generally Recognized as Safe
HDAC: histone deacetylase
5-HT: 5-hydroxytryptamine (serotonin)
IDH: isocitrate dehydrogenase
IDO: indoleamine 2,3-dioxygenase
IL-1β, IL-4, IL-6, IL-10, IL-23: interleukin-1β, interleukin-4, interleukin-6, interleukin-10, interleukin-23
KYNA: kynurenic acid
LPS: lipopolysaccharide
m^6^A: N6-methyladenosine
MR: Mendelian randomization
*MYC*: *MYC* proto-oncogene
NCS: non-caloric sweetener(s)
NF-κB: nuclear factor kappa-light-chain-enhancer of activated B cells
NHDC: neohesperidin dihydrochalcone
NLRP6: NOD-like receptor family, pyrin domain containing 6
OD: optical density
PD-1/PD-L1: programmed cell death protein 1/its ligand
PGE2: prostaglandin E2
PI3K/AKT: phosphoinositide 3-kinase/protein kinase B
PPAR-γ: peroxisome proliferator-activated receptor gamma
ROS: reactive oxygen species
SCFA: short-chain fatty acid
T1R2/T1R3: taste receptor type 1, members 2 and 3
TCDCA: taurochenodeoxycholic acid
TCGA: The Cancer Genome Atlas
*TGFB1/TGFB2*: transforming growth factor beta 1/2
TiO_2_ (E171): titanium dioxide
TLR4: Toll-like receptor 4
TNF-α: tumor necrosis factor alpha
TRAF6: TNF receptor-associated factor 6
XAI: explainable artificial intelligence
ZO-1 (*Tjp1*): zonula occludens-1 (tight junction protein 1)
